# Supplementary figures and images for: Genetic analyses reveal population structure and recent decline in leopards (Panthera pardus fusca) across the Indian subcontinent
Source: PeerJ. 2020 Feb 4;8:e8482. doi: 10.7717/peerj.8482 (PMC7006512; doi:10.7717/peerj.8482)

$$\text{DeltaK} = \text{mean}(|L''(K)|) / \text{sd}(L(K))$$

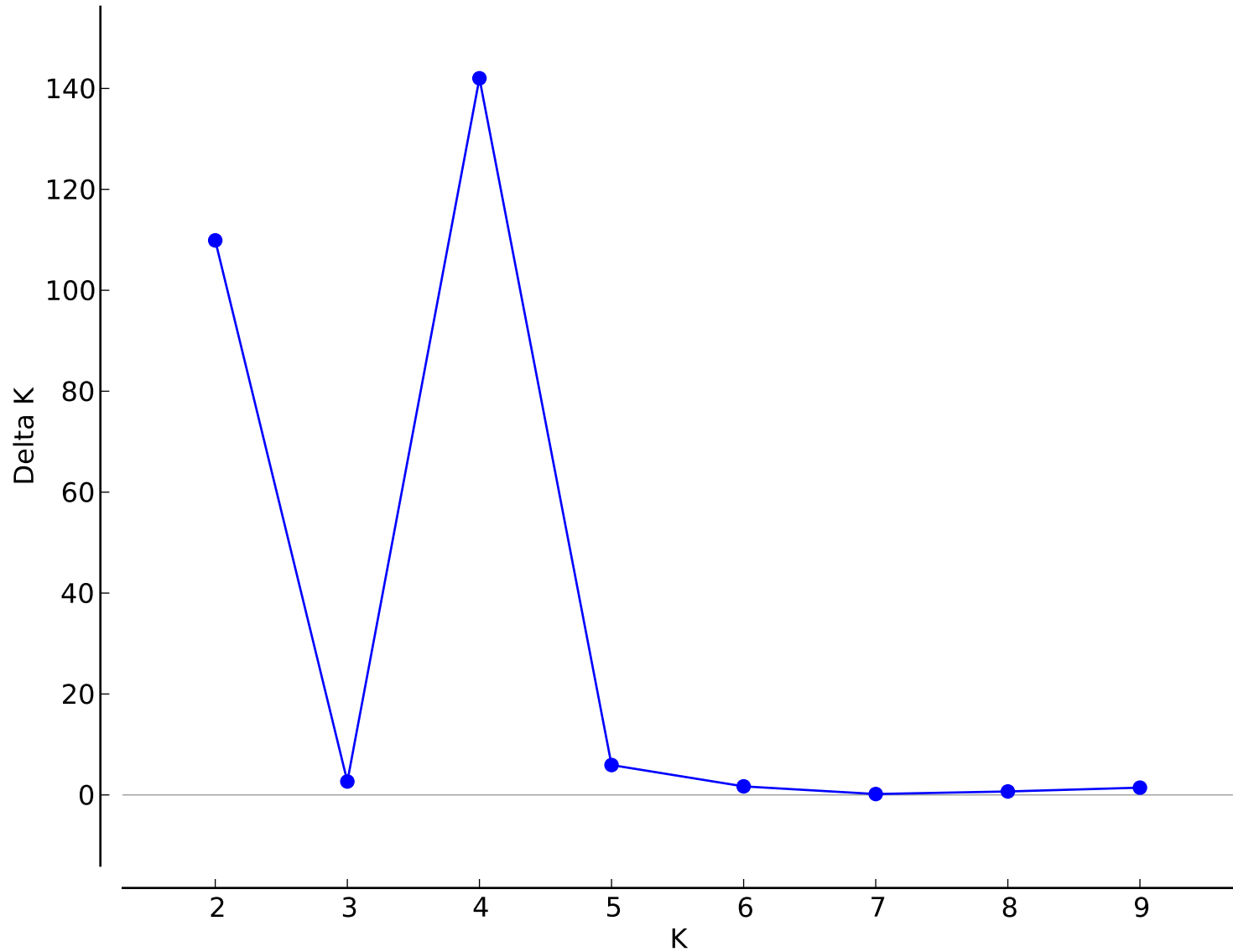

Supplement: Figure S1 [file peerj-08-8482-s001.pdf]

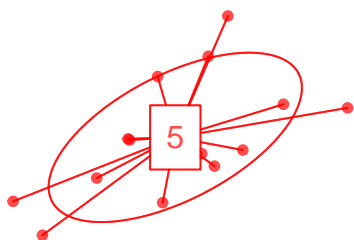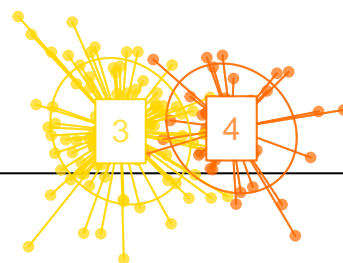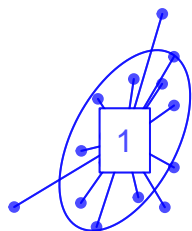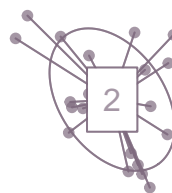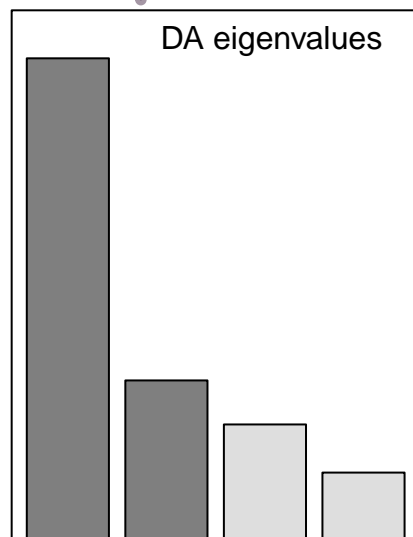

Supplement: Figure S2 — Results indicate five different genetic subpopulations, where two of them are overlapping compared to the other clusters. [file peerj-08-8482-s002.pdf]
