# Supplementary material for: Genetic analyses reveal population structure and recent decline in leopards (Panthera pardus fusca) across the Indian subcontinent
Source: PeerJ. 2020 Feb 4;8:e8482. doi: 10.7717/peerj.8482 (PMC7006512; doi:10.7717/peerj.8482)
Supplement: Table S1 — A generation time of 5 years is used. [file peerj-08-8482-s003.pdf]

Supplementary Table 1– Prior models used for leopard demography analyses in Storz and Beaumont method. A generation time of 5 years is used.

**Runs**	**log(N_0)**	**log(N_1)**	**log(\)**	**log(T)**	**log(N_0)**	**log(N_1)**	**log(\)**	**log(T)**
**Run 01**	**5 1**	**5 1**	**-3.5 1**	**3 1**	**3.5 2 0 0.5**	**6 3 0 0.5**	**-3.5 0.25 0 0.5**	**3 2 0 0.5**
**Run 02**	**5 1**	**5 1**	**-3.5 1**	**3 1**	**3.5 2 0 0.5**	**5 2 0 0.5**	**-3.5 0.25 0 0.5**	**3 2 0 0.5**
**Run 03**	**5 1**	**5 1**	**-3.5 1**	**3 1**	**3.5 2 0 0.5**	**5 1 0 0.5**	**-3.5 0.25 0 0.5**	**3 2 0 0.5**
**Run 04**	**5 1**	**5 1**	**-3.5 1**	**3 1**	**3.5 2 0 0.5**	**6 1 0 0.5**	**-3.5 0.25 0 0.5**	**3.5 2 0 0.5**
**Run 05**	**5 1**	**5 1**	**-3.5 1**	**3 1**	**4 2 0 0.5**	**6 2 0 0.5**	**-3.5 0.25 0 0.5**	**3 2 0 0.5**
**Run 06**	**5 1**	**5 1**	**-3.5 1**	**3 1**	**3.5 2 0 0.5**	**5 1 0 0.5**	**-3.5 0.25 0 0.5**	**3 2 0 0.5**
